# The Friendship Questionnaire, autism, and gender differences: a study revisited

**DOI:** 10.1186/s13229-019-0295-z

**Published:** 2019-11-28

**Authors:** Felicity Sedgewick, Jenni Leppanen, Kate Tchanturia

**Affiliations:** 1 Psychological Medicine, Institute of Psychiatry, Psychology and Neuroscience, King’s College London, London, SE5 8AF UK; 2 South London and Maudsley NHS Foundation Trust National Eating Disorder Service, London, UK; 3 Psychology Department, Illia State University, Tbilisi, Georgia; 4 School of Education, University of Bristol, Bristol, UK

**Keywords:** Autism, Gender, Non-binary, Friendship, Relationship, Social communication

## Abstract

**Background:**

The Friendship Questionnaire (FQ) is a widely used measure of friendships in autism research and beyond. This study sought to revisit the original paper where the measure was presented, using a larger sample of both autistic and non-autistic participants to examine gender differences in scoring. It also sought to expand upon the original paper by comparing FQ results to those of the Unidimensional Relationship Closeness Scale (URCS), to examine whether there are differences in how autistic people report on their general friendships in contrast to their most significant relationships.

**Methods:**

Participants were recruited for an online study, and 949 people (532 autistic, 417 non-autistic) aged between 18 and 81 took part. Participants completed a demographic questionnaire, the Autism Quotient-28, the Friendship Questionnaire, and the Unidimensional Relationship Closeness Scale.

**Results:**

We used robust regressions and Pearson’s correlational analyses, conducted in R. Autistic people scored lower than non-autistic people on the FQ, and similar gender differences in the pattern of FQ scores were seen in both groups. There was a significant negative correlation between AQ and FQ scores in both groups. On the URCS, we took the data from those who rated specific close relationships and found that autistic people scored this relationship more highly than non-autistic adults did. There was a significant negative correlation between AQ and URCS scores in both groups. Also, in both groups, there was a significant positive correlation between FQ and URCS scores.

**Limitations:**

The data is entirely self-report, and diagnoses could not be verified with a clinician, although AQ scores support self-identification as autistic. Also, the groups were not evenly matched on age and other demographic variables, although this was controlled for in analyses. It is also the case that more autistic than non-autistic people were unable to specify a close relationship to score on the URCS, meaning that a certain set of experiences are not represented in this data.

**Conclusions:**

We conclude that our data replicates the core finding of the original FQ paper that autistic people score lower on the FQ. In contrast to that paper, however, we found that there were gender differences among the autistic population. Also, our inclusion of the URCS suggests that the intimate romantic relationships and best-friendships of autistic people can be of similar quality to those of non-autistic people, suggesting that there may be important differences in autistic people’s relations with friends in general versus close friends and romantic partners.

## Introduction

In 2003, Baron-Cohen and colleagues created, tested, and published a measure of friendship quality called the Friendship Questionnaire [1]. In this initial study, the authors found that autistic adults (51 males, 17 females) scored lower on their 35-item questionnaire about friendships, social behaviours, and social cognitions than non-autistic adults (27 males, 49 females), which they took to represent lower quality friendships among autistic people and to suggest that there were no gender differences in friendship quality among autistic adults. Since the initial publication, however, there have been no explicit replication studies of this paper, despite widespread use of the measure [2–5].

There is a common perception that some autistic children, young people, and adults do not *want* to have friends [6] as difficulties in the social realm are diagnostic criterion for autism [7]. Some research has found that autistic children have fewer friendships [8], are more lonely [9], and are more socially excluded than both non-autistic peers and peers with other developmental conditions [10]. Review studies have suggested that autistic children had lower friendship reciprocity, spend less time with the friends they do have, and have lower quality friendships than non-autistic children [11]. Autistic adolescents have been shown to often experience social isolation and concomitant peer victimisation and mental health issues [12]. Social networking studies have consistently shown that autistic students are less accepted and included by their peers in the classroom, across all stages of school [10, 13, 14]. These methods, however, are often focussed on the number and structure of friendships young people have, rather than the quality of a small number of connections. Adult outcome studies have found that autistic people are less likely to be married or in a long-term romantic relationship [15, 16] and are more likely to rely on their parents for social support than on same-age peers or colleagues [17].

More recent research, however, has suggested that many autistic children, young people, and adults desire, have, and maintain successful friendships and romantic relationships [6, 18–20]. While there are still challenges in friendships for autistic people, making friends who accept and normalise their autism was crucial [21]. Qualitative research has shown that these relationships are often credited as crucial factors in wider success in life [22], just as social support is key for non-autistic people [23, 24]. Interview studies with autistic young women have revealed that making and maintaining friendships are important to this group [25, 26], potentially more so than it is for young men [20, 27]. Furthermore, despite parent and professional assumptions that many young people will not be interested in or aware of romantic/sexual relationships [28, 29], autistic young people and adults are often more knowledgeable and active than others realise [30–33].

These papers, however, have tended to have small sample sizes, or to focus on young people, or to only include a single gender of participants. This means that there is a gap in the literature both in terms of evaluating the FQ, where the measure is often used unquestioningly rather than being compared to other measures or linked to qualitative discussions, and also in terms of understanding potential gender differences in the relationships of autistic adults, something which is crucial in designing effective support strategies for those who need them. What little work has studied gender differences in the relationships of autistic people has focussed on children and adolescents and has suggested that autistic girls are likely to have stronger best-friendships than autistic boys [19, 20], and experience higher levels of different types of victimisation, being more neglected than actively rejected by their peers [5]. One paper which has recently looked at the friendship experiences of 18–24-year-old autistic adults found some differences between men and women in terms of what predicted their friend choice and friendship styles [34], suggesting that gender differences seen in non-autistic populations may also be present in autistic people.

No research claims that autistic people do not face challenges with their social relationships, and indeed often discusses the difficulties autistic individuals experience. Much work has highlighted struggles autistic people have with being bullied [35–37], for example, and victimisation can continue into adulthood [38], with social difficulties also impacting on the ability to find and stay in employment [39]. Despite these difficulties, it is important to recognise and study the many positive social experiences autistic people have, as these are just as important as any challenges they face.

This paper sought to replicate the findings from Baron-Cohen and colleagues’ original 2003 paper, along with expanding the remit by including non-binary and transgender people (NBT) (those who identify with neither or both male and female gender characteristics, regardless of their gender assigned at birth, here including transgender people for statistical purposes) who have recently been shown to make up a large portion of the autistic community [40, 41]. It is likely that NBT people, both autistic and non-autistic, have a unique set of relationship experiences and influences which have not to date been explored in research. The explicit inclusion of this so-far ignored population in our work, therefore, represents an entirely novel and important contribution to the literature, beginning to describe the experiences of a group who may constitute as much as a third of the autistic population.

We also sought to extend the study by asking participants to complete the Unidimensional Relationship Closeness Scale (URCS) on which they rated a nominated relationship, allowing for examination of specific relationships as well as the wider friendships assessed by the FQ. Considering the little extant work on the relationships of autistic adults, which has varied findings—from autistic adults being unlikely to have friends [42] to autistic women having difficulties with casual social relationships such as work colleagues or school-gate chat (something as yet unstudied in autistic men) [19, 39]—we predicted that just as autistic people tend to score their wider social relationships lower on the FQ, they would score their intimate relationship lower on the URCS.

Our hypotheses were that:

1. Autistic participants would score lower on the FQ than non-autistic participants
2. Gender would have a significant impact on FQ score in both autistic and non-autistic participants, with women and NBT people expected to have higher scores as compared to men in line with qualitative research on the friendships of autistic adults
3. Autistic participants would score lower on the URCS than non-autistic participants, similar to their scoring patterns on the FQ
4. Gender would have a significant impact on URCS score in both groups, with women and NBT people expected to have higher scores than men
5. There will be a significant negative correlation between autism quotient (AQ) scores and the FQ and URCS questionnaires, such that autism symptomatology increases, FQ and URCS scores decrease

Interactions were tested as secondary, exploratory research questions for all measures.

## Method

### Participants

Nine hundred and thirty-one people between the ages of 18 and 81 were included in the analysis, after the exclusion of 14 participants who reported being below 18 and therefore did not meet the age criteria for inclusion. A further 18 participants were excluded for scoring over 21 on the AQ but reporting being non-autistic, in order to retain clear boundaries between the groups. All participants who reported having an autism diagnosis were retained in the sample regardless of AQ score, as it has been recognised that the AQ may not be as sensitive to autistic traits in non-male groups [43]. Of the remaining 931 participants, 532 (57.1%) reported that they were autistic, and 391 (41.9%) reported no autism diagnosis. Demographic characteristics can be seen in Table 1.

**Table 1:** Demographic characteristics of the sample by group (autistic, non-autistic) and gender (male, female, NBT). Ethnicity, education level, and employment are whole-sample values

	Autistic (*n* = 532)			Non-autistic (*n* = 391)		
	Male (*n* = 72)	Female (*n* = 317)	NBT (*n* = 143)	Male (*n* = 54)	Female (*n* = 327)	NBT (*n* = 18)
Age						
Range, M (SD)	18.09–71.42, 36.04 (14.37)	18.12–71.53, 35.18 (11.22)	18.10–57.00, 28.85 (8.00)	18.95–65.98, 33.08 (10.97)	18.15–81.29, 31.79 (10.54)	18.29–57.24, 30.24 (9.39)
AQ score						
Range, M (SD)	6–28, 20.61 (4.21)	3–27, 21.13 (3.91)	9–27, 21.22 (3.34)	1–20, 10.33 (4.88)	0–20, 8.74 (5.28)	0–20, 12.67 (6.48)
Ethnicity						
White		393 (74.01%)			307 (76.94%)	
Asian		10 (1.88%)			25 (6.26%)	
Black		4 (0.75%)			4 (1.01%)	
Latinx		5 (0.94%)			2 (0.50%)	
Mixed		29 (5.46%)			13 (3.26%)	
No answer		90 (16.95%)			50 (12.52%)	
Education level						
None		23 (4.33%)			2 (0.50%)	
GCSE		20 (3.76%)			5 (1.25%)	
A-level		83 (15.63%)			35 (8.77%)	
Diploma		62 (11.67%)			15 (3.76%)	
Bachelors		205 (38.61%)			170 (42.61%)	
Masters		111 (20.90%)			133 (33.33%)	
PhD		24 (4.52%)			37 (9.27%)	
No answer		3 (0.56%)			2 (0.50%)	
Employment						
Full-time		135 (25.42%)			210 (52.63%)	
Part-time		67 (12.62%)			36 (9.02%)	
Student		109 (20.53%)			108 (27.07%)	
Self-employed		57 (10.73%)			17 (4.26%)	
Unemployed		101 (19.02%)			14 (3.51%)	
Retired		11 (2.07%)			4 (1.01%)	
Other		48 (0.87%)			10 (2.50%)	
No answer		3 (0.56%)			2 (0.50%)	

Seventeen individuals identified themselves as transgender, 14 autistic people, and 3 non-autistic people. Testing revealed that their responses were not significantly different to those of other non-binary participants, and so they were included in the over-arching NBT group, rather than removing their data entirely. This would have been necessary if trying to treat them as a separate group, as the numbers involved are too small for valid statistical comparisons to be made to other groups.

Participants were recruited online through social media (Twitter, Facebook) and through online advertising on the King’s College website and email circulars. Ethical approval was obtained from the King’s Psychiatry, Nursing and Midwifery Research Ethics Committee (LRS-17/18-5292). All participants read a full information page before taking part in the study, completed an informed consent page after reading the information page, and were further informed that completing the study would be taken as consent for the use of their data. All procedures were conducted in accordance with the latest version of the Declaration of Helsinki.

### Measures

#### Demographics

Participants completed a demographics questionnaire, including their age, autism status, ethnicity, and employment status.

#### AQ

The Autism Quotient-28 item version [44] is a self-report screening questionnaire assessing the presence and level of autism symptomatology an individual experiences. Answers are given on a 4-point Likert scale from ‘Definitely agree’ to ‘Definitely disagree’ and are then scored 1 or 0 depending on the direction of the question. Example items include ‘I prefer to do things with others rather than on my own’ and ‘I am fascinated by dates’. Higher scores reflect higher levels of autistic symptomatology. A cut-off of 21 was used for likely autism, in line with calculations by the original authors [25]. The AQ was used because it is simple for participants to understand, is shorter than many other screening measures, and, despite some recognised issues described later in the manuscript, is generally well-validated. Cronbach’s alpha in this sample was 0.87 for non-autistic and 0.94 for autistic participants.

#### FQ

The Friendship Questionnaire [1] is a 35-item questionnaire, of which 27 items are scored either 0, 2, or 5, with 5 being a maximum score. Participants are asked to decide which of the three options describe them best, with each one being assigned a score the participant cannot see, for example—(a) I have one or two particular best friends (5), (b) I have several friends who I would call best friends (2), and (c) I don’t have anybody who I would call a best friend (0). Answers are summed for a maximum score of 135. Higher scores are reflective of better or more friendships. Cronbach’s alpha in this sample was 0.71 for autistic and 0.84 for non-autistic participants.

#### URCS

The Unidimensional Relationship Closeness Scale [45] is a 12-item self-report questionnaire which asks participants to rate features of their closest relationship on a 7-point Likert scale ranging from 1 (strongly disagree) to 7 (strongly agree). Example items include ‘My relationship with ____ is close’ and ‘I consider my ___ when making important decisions’. Scores are then calculated by averaging across all 12 items. The URCS measures how close their relationship is, with a population mean score of 6.00 for romantic couples and 5.02 for same-sex friends [45]. Higher scores reflect greater closeness in the nominated significant relationship. In this study, participants were asked to categorise the relationship they were describing (0 = long-term romantic partner; 1 = dating romantic partner; 2 = best friend; 3 = family; 4 = other). Following the group differences identified by Dibble et al., those who answered for long-term or dating romantic partners were collapsed into one group, with their results compared to those who answered for a best friend. There were 26 participants who did not feel they had a close relationship, of whom 20 were autistic, and they left this portion of the survey blank or gave responses of 0 to each question. Their results were excluded from analyses. Cronbach’s alpha was 0.75 for autistic and 0.83 for non-autistic participants.

### General procedure

Participants all completed the study online, at their own pace and in a place of their preference. The data were collected as part of a larger study. Participants completed the demographic information, the AQ, the FQ, and the URCS in that order. The survey was delivered using onlinesurveys.ac.uk. Each questionnaire was on a separate survey page, with instructions to participants to select the answer options which were most true for or best described them. Each question was written out in full before the answer options were presented, and questions ran sequentially down the page. Once a measure was complete, participants clicked through to the next page which contained the next questionnaire.

### Data analysis

All data analyses were conducted with R [46]. Group differences in demographic and clinical characteristics were explored with *t* tests. Due to differences in group size, robust M-estimator was used to assess group differences [47–49]. When examining group differences in FQ scores, autism spectrum (AS) status (autistic, non-autistic) and gender (male, female, NBT) were included as predictors. The model residuals from the robust M-estimators were then visually inspected, and studentized Breusch-Pagan test was conducted to ensure no significant heteroscedasticity was present. When examining group differences in the URCS scores, the present study focused on those participants who rated their best friend. Group differences in URCS scores were examined using a robust M-estimator with AS status (autistic, non-autistic) and gender (male, female, NBT) as predictors. As with the FQ scores, the model residuals were visually inspected and studentized Breusch-Pagan test was conducted to ensure no significant heteroscedasticity was present. Significant main effects and interactions were explored with post hoc pairwise comparisons. Pearson’s correlation analyses were conducted to examine correlations between AQ scores and FQ and URCS scores in each group. Hedge’s *g* was used to calculate the effect sizes for all analyses. The effect sizes were interpreted as small, 0.2 ≤ *g* < 0.5; medium, 0.5 ≤ *g* < 0.8; or large, *g* ≥ 0.8 [50]. To avoid using an arbitrary cut-off for statistical significance, all significance tests in the present study were subjected to false discovery rate correction for multiple comparisons (*q* = 0.05) [51]. *P* values less than 0.039 were considered statistically significant.

Due to significant differences in age across groups, this was not added as a covariate to the main analyses examining differences in friendship score and relationship closeness across the autism spectrum. Including variables that are highly correlated or have a significant relationship as predictors introduced multicollinearity potentially leading to false-negative findings [52].

## Results

### Demographics

Participants were not matched on age, *t*(930) = − 2.35, *p* = 0.02, *g* = − 0.14, 95% CI [− 0.27, − 0.01], with autistic participants being older than non-autistic participants. Participants were also not matched on AQ score, with those who reported being autistic scoring significantly higher than those who reported being non-autistic, *t*(930) = − 37.93, *p* < 0.001, *g* = − 2.33, 95% CI [− 2.50, − 2.17].

### Friendship Questionnaire

Scores on the FQ can be seen in Table 2. A 2 (autism status) by 3 (gender) robust M-estimator was conducted on FQ scores. The residual plots (Additional file 1: Figure S1) and the studentized Breusch-Pagan test did not reveal significant heteroscedasticity (BP(5) = 2.65, *p* = 0.754). There was a main effect of autism status, *F*(1) = 34.67, *p* < 0.001, autistic: mean = 56.15, SD = 17.89; non-autistic: mean = 79.09, SD = 18.98; *g* = 1.25, 95% CI [1.11, 1.39], and a main effect of gender, *F*(2) = 13.14, *p* < 0.001; male: mean = 59.37, SD = 20.65; female: mean = 68.58, SD = 22.27; NBT: mean = 60.73, SD = 17.09; male vs. female: *g* = − 0.42, 95% CI [− 0.61, − 0.23]; male vs. NBT: *g* = − 0.07, 95% CI [0.16, − 0.31]; female vs. NBT: *g* = 0.37, 95% CI [0.19, 0.54]. There was a significant interaction between autism status and gender, *F*(2) = 8.74, *p* < 0.001, such that autistic NBT scored higher than autistic men, *z* = 3.55, *p* = 0.001, *g* = 0.47, 95% CI [0.19, 0.76], and autistic women, *z* = 2.46, *p* = 0.037; *g* = 0.22, 95% CI [0.02, 0.42]. There were no significant differences between autistic men and autistic women, *z* = 2.04, *p* = 0.104; *g* = 0.28, 95% CI [0.02, 0.53]. Among the non-autistic participants, men scored significantly lower than women, *z* = − 4.21, *p* < 0.001; *g* = − 0.57, 95% CI [− 0.86, − 0.28], and women scored significantly higher than NBT participants, *z* = 3.27, *p* = 0.003; *g* = 0.74, 95% CI [0.26, 1.22]. There was no significant difference between non-autistic men and non-autistic NBT participants, *z* = 0.64, *p* = 0.801; *g* = − 0.18, 95% CI [− 0.71, 0.35]. Post hoc pairwise comparisons between autistic and non-autistic participants within each gender group revealed that non-autistic men scored significantly higher than autistic men, *z* = 5.89, *p* < 0.001; *g* = 1.08, 95% CI [0.69, 1.45], as did non-autistic women when compared to autistic women, *z* = 17.93, *p* < 0.001; *g* = 1.38, 95% CI [1.21, 1.55]. There was no significance in the FQ score difference between autistic and non-autistic NBT participants, *z* = 1.50, *p* = 0.135; *g* = 0.39, 95% CI [− 0.10, 0.88].

**Table 2:** FQ scores by group (autistic, non-autistic) and gender (male, female, NBT)

	Autistic (*n* = 532)			Non-autistic (*n* = 391)		
	Male (*n* = 72)	Female (*n* = 317)	NBT (*n* = 143)	Male (*n* = 54)	Female (*n* = 317)	NBT (*n* = 18)
FQ score						
Range	5 99	16–111	23–102	36–109	21–120	40–98
M (SD)	50.93 (18.63)	55.93 (17.93)	59.90 (18.97)	70.62 (17.75)	81.13 (18.59)	67.33 (19.09)

There was a significant negative correlation between FQ score and AQ score in both the autistic, *r* = − 0.38, *p* < 0.001, and non-autistic, *r* = − 0.56, *p* < 0.001, groups. This meant that as AQ score rose, FQ score dropped regardless of whether participants were autistic or not.

### Unidimensional Relationship Closeness Scale

When examining scores on the URCS, we selected data from only those participants who had nominated a married/live-in partner, dating partner, or best friend in answering the questions, as these are the relationships with established population norms according to the original authors of the measure. This left a total of 470 autistic participants and 405 non-autistic participants, representing 92.20% of the original sample. The 2 (autism status) by 3 (gender) robust M-estimator model showed significant heteroscedasticity (Additional file 2: Figure S2), studentized Breusch-Pagan test BP(5) = 24.67, *p* < 0.001. The level of heteroscedasticity was too substantial to adjust with data transformation or more robust statistics. Therefore, the URCS sample was split into two smaller samples which were analysed separately. The first sample included participants who rated their best friend or dating partner, and the second sample included participants who rated their married or live-in partner. Those who rated a dating partner and a best friend were combined because these groups have been found to have similar population norm scores (5.00 and 5.02 respectively), compared to a norm of 6.00 for married couples [44]. The first URCS sample included 227 autistic and 181 non-autistic people, while the second URCS sample included 243 autistic and 208 non-autistic people. Participant demographics and their URCS scores are described in Table 3.

**Table 3:** Demographics and questionnaire scores by group (autistic, non-autistic) and gender (male, female, NBT)

	Autistic (*n* = 227)			Non-autistic (*n* = 181)		
	Male (*n* = 28)	Female (*n* = 147)	NBT (*n* = 78)	Male (*n* = 23)	Female (*n* = 121)	NBT (*n* = 11)
Age						
Range	18.66–64.94	18.12–65.28	19.24–52.92	20.79–62.79	18.15–67.23	18.68–42.15
M (SD)	34.38 (15.56)	31.63 (10.94)	26.43 (6.81)	29.12 (9.17)	28.57 (8.64)	26.46 (6.42)
AQ score						
Range	6.00–27.00	3.00–27.00	13.00–27.00	1.00–15.00	0.00–20.00	4.00–21.00
M (SD)	19.93 (4.68)	21.12 (3.85)	21.37 (3.17)	10.04 (4.02)	9.13 (5.35)	13.73 (6.72)
FQ score						
Range	27.00–99.00	23.00–111.00	23.00–102.00	55.00–89.00	51.00–115.00	45.00–98.00
M (SD)	56.50 (19.82)	59.26 (17.90)	62.03 (16.41)	69.13 (16.23)	80.44 (16.22)	60.55 (16.26)
URCS score						
Range	1.17–6.75	0.50–7.00	1.33–7.00	1.33–6.92	0.83–7.00	1.50–6.50
M (SD)	4.46 (1.75)	5.01 (1.45)	5.42 (1.40)	5.43 (1.64)	5.56 (1.25)	3.94 (2.01)

#### Unidimensional Relationship Closeness Scale—best friends/dating partners

A 2 (autism status) by 3 (gender) robust M-estimator was conducted to examine differences on URCS scores in the first sample of people who rated their best friend or dating partner. The residual plots (Additional file 3: Figure S3) and the studentized Breusch-Pagan test did not reveal significant heteroscedasticity (BP(5) = 6.49, *p* = 0.262). There was a significant main effect of autism status on URCS score, *F*(1) = 7.34, *p* = 0.007, autistic: mean = 5.08, SD = 1.50; non-autistic: mean = 5.45, SD = 1.40, *g* = 0.25, 95% CI [0.05, 0.45]. There was a significant main effect of gender, *F*(2) = 5.20, *p* = 0.0060, male: mean = 4.90, SD = 1.76; female: mean = 5.31, SD = 1.37; NBT: mean = 5.24, SD = 1.55; male vs. female *g* = − 0.28, 95% CI [− 0.58, 0.02]; male vs. NBT: *g* = − 0.21, 95% CI [− 0.54, 0.12]; female vs. NBT: *g* = 0.05, 95% CI [− 0.19, 0.29]. There was also a significant interaction between autism status and gender, *F*(2) = 8.91, *p* < 0.001, such that autistic men scored significantly lower than autistic NBT participants, *z* = − 3.05, *p* = 0.006; *g* = − 0.64, 95% CI [− 1.08, − 0.20]. There was no significant difference between autistic women and autistic NBT participants, *z* = − 2.19, *p* = 0.073; *g* = − 0.29, 95% CI [− 0.56, − 0.009], or between autistic men and autistic women, *z* = − 1.69, *p* = 0.209; *g* = 0.36, 95% CI [− 0.04, 0.77]. Among the non-autistic participants, both women and men scored significantly higher than NBT participants, *z* = 3.20, *p* = 0.004; *g* = 1.22, 95% CI [0.58 1.85], and *z* = 2.79, *p* = 0.015; *g* = 0.82, 95% CI [0.08, 1.57], respectively. There were no significant differences between non-autistic men and non-autistic women, *z* = 0.09, *p* = 0.995; *g* = 0.10, 95% CI [− 0.35, 0.54]. Post hoc pairwise comparisons between autistic and non-autistic participants within each gender group revealed that autistic NBT participants scored significantly higher than non-autistic NBT participants, *z* = 2.89, *p* = 0.004; *g* = 1.00, 95% CI [0.34, 1.64]. The opposite pattern was seen among men and women, such that autistic men scored significantly lower than non-autistic men, *z* = − 2.71, *p* = 0.007; *g* = − 0.56, 95% CI [− 1.12, 0.001], and autistic women scored significantly lower than non-autistic women, *z* = − 3.15, *p* = 0.002; *g* = − 0.40, 95% CI [− 0.65, − 0.16].

There was a significant negative correlation between URCS score and AQ score in the autistic, *r* = − 0.23, *p* < 0.001, and non-autistic, *r* = − 0.32, *p* < 0.001, groups, such that higher AQ scores were associated with lower URCS scores.

There was also a significant positive correlation between FQ score and URCS score in both the autistic, *r* = 0.46, *p* < 0.001, and non-autistic, *r* = 0.30, *p* < 0.001, groups, such that higher FQ scores were associated with higher URCS scores.

#### Unidimensional Relationship Closeness Scale—married/live-in partners

Participant characteristics for those who scored a married/live-in partner are presented in Table 4. A 2 (autism status) by 3 (gender) robust M-estimator was conducted to examine differences on URCS scores in the second sample of people who rated their married or long-term partner. The URCS scores in this sample required further logarithm transformation to meet the homoscedasticity assumptions (BP(5) = 7.57, *p* = 0.182; Additional file 4: Figure S4). There was no significant main effect of autism status, *F*(1) = 1.28, *p* = 0.258, autistic: mean = 5.89, SD = 1.34; non-autistic: mean = 6.26, SD = 1.03; *g* = 0.31, 95% CI [0.12, 0.49], or that of gender, *F*(2) = 0.61, *p* = 0.543, male: mean = 6.01, SD = 1.13; female: mean = 6.05, SD = 1.26; NBT: mean = 6.15, SD = 1.11; male vs female: *g* = − 0.03, 95% CI [− 0.31, 0.24]; male vs. NBT: *g* = − 0.12, 95% CI [− 0.48, 0.23]; female vs. NBT: *g* = − 0.08, 95% CI [− 0.35, 0.19]. There was also no significant gender by autism status interaction, *F*(2) = 0.78, *p* = 0.459.

**Table 4:** Demographics and questionnaire scores by group (autistic, non-autistic) and gender (male, female, NBT)

	Autistic (*n* = 243)			Non-autistic (*n* = 208)		
	Male (*n* = 31)	Female (*n* = 155)	NBT (*n* = 57)	Male (*n* = 29)	Female (*n* = 172)	NBT (*n* = 7)
Age						
Range	18.57–71.42	18.53–71.53	19.71–57.00	20.27–65.98	20.95–68.25	22.30–57.24
M (SD)	35.99 (12.72)	38.42 (10.20)	31.76 (8.58)	35.37 (11.47)	34.35 (10.65)	36.19 (10.68)
AQ score						
Range	13.00–27.00	6.00–27.00	9.00–26.00	2.00–19.00	0.00–20.00	0.00–19.00
M (SD)	21.32 (3.73)	21.43 (3.68)	21.32 (3.46)	10.17 (5.43)	8.30 (5.16)	11.00 (6.22)
FQ score						
Range	28.00–92.00	16.00–101.00	27.00–95.00	36.00–109.00	27.00–120.00	44.00–94.00
M (SD)	51.29 (16.61)	54.32 (17.59)	59.21 (16.19)	72.17 (19.48)	82.99 (19.44)	78.00 (19.36)
URCS score						
Range	2.25–7.00	1.33–7.00	1.08–7.00	3.50–7.00	2.00–7.00	2.33–7.00
M (SD)	5.80 (1.30)	5.79 (1.44)	6.20 (1.05)	6.23 (0.89)	6.28 (1.03)	5.82 (1.61)

There were significant negative correlations between URCS and AQ scores in the married autistic, *r* = − 0.13, *p* = 0.036, and married non-autistic, *r* = − 0.20, *p* = 0.005, groups. There were also significant positive correlations between URCS and FQ scores in the married autistic, *r* = 0.17, *p* = 0.009, and married non-autistic, *r* = 0.30, *p* < 0.001, groups.

## Discussion

This study aimed to replicate findings on the presence, or lack, of gender differences in friendships of Baron-Cohen and colleagues’ original 2003 paper. Overall, our analysis shows that autistic adults reported having positive, close, and supportive relationships. The patterns on the FQ to an extent replicated those from the original study [1], and the correlations between higher AQ scores and lower scores on both the FQ and the URCS further support the original conclusions that those on the autism spectrum are likely to rate their friendships lower than those who are not autistic. Interestingly, however, the URCS revealed that autistic adults scored their nominated relationships as more emotionally close than non-autistic adults, a finding we discuss in detail below.

### Comparison to results from Baron-Cohen et al. [1]

In comparison to the results reported in the original Baron-Cohen et al. paper [1], the patterns seen between men and women, and autistic and non-autistic people in this study were very similar in many ways, although with some points of difference.

Baron-Cohen et al. found that non-autistic women scored significantly higher than non-autistic men, but that there were no significant differences between autistic men and women. They also found that autistic people scored significantly lower on the FQ than non-autistic people. We also found that autistic people scored lower than non-autistic people on the FQ, with a large effect size emphasising the importance of this finding, but we found that there was a significant impact of gender on the FQ score. This effect was such that NBT autistic people scored more highly than autistic men and women, with small effect sizes. Autistic women scored more highly than men, although this did not quite reach our stringent statistical significance level. This finding is in line with a wealth of work on gender differences in friendships among non-autistic people [4, 53]. It also echoes recent findings of gender differences in friendship ratings and experiences among autistic children and young people [18, 20, 27, 54]. Taken together, these findings suggest that there are likely to be gender differences in the friendships of autistic people, in contrast to the findings of the original paper.

Our study extended the original study by including NBT individuals, reflecting the growing recognition that NBT gender identities are prevalent in the autistic population [40, 41]. In the non-autistic group, NBT people scored significantly lower on the FQ than women and similarly to men—though this should be interpreted with caution considering the very small numbers of respondents. In the autistic group, by contrast, NBT people scored highest on the FQ, contributing to the significant interaction between autism and gender. This is an interesting update to the conclusions of Baron-Cohen et al. [1] and maybe because those of non-traditional gender identities are required to do more conscious work in navigating their relationships [55]. This greater openness and discussion may lead to closer relationships along with the stigma non-gender-conforming individuals can often face [56].

It is worth noting that the average scores of non-autistic women in this study (81.13) were lower than the average scores of the non-autistic women in the Baron-Cohen et al. paper (90.00). This is possibly due to larger sample size in the present study. The scores of our autistic men and women (men 50.93; women 66.93) were like those of the initial paper (men 53.20; women 59.80). Interestingly, this means that the average scores of the autistic participants in this study are in what the original authors considered the ‘low’ range, as are the scores of our non-autistic male and NBT participants. This suggests that perhaps the initial delineation of ‘low’, ‘middle’, and ‘high’ ranges would benefit from revision, as currently the majority of all people completing the questionnaire are categorised as ‘low’, rather than meeting criteria for ‘middle’ scores. If, following the principles of normal distribution, we equate the ‘middle’ range with where we would expect the majority of participants to fall, our findings suggest that the ranges should be moved down somewhat, although the nature of these changes would require explicit investigation.

### URCS and relationship to FQ

The results from the URCS somewhat complicate the picture from the FQ and the original paper, though it should be noted that they are not comparable to those of the original study in the strictest sense, as the URCS focusses on close rather than general relationships. Significant correlations between the URCS and the FQ suggest that these two measures may tap similar constructs, and the similar relationships between both measures and the AQ show that as general friendship ratings (FQ score) increase, so do ratings of a close relationship (URCS), and that as autistic symptomatology increases, relationship ratings decrease.

Dividing the sample into those who rated their best friend/dating partner and those who rated a married/long-term partner revealed fascinating differences in the impact of autism status on these relationships. Among those who rated a best friend/dating partner, we saw a similar pattern on the URCS to that on the FQ that autistic people gave lower scores to this relationship than non-autistic people, though the small effect size suggests that this should be interpreted with caution. Despite this caution, this finding is to be expected, considering the wealth of work which shows that autistic people are likely to experience significant challenges in their broader social relationships [13, 14, 39]. There was, however, no consistent effect of gender, with different patterns emerging in the autistic and non-autistic groups, possibly due to the disparity in numbers of NBT people in each sample.

In contrast, among those who rated a married/long-term partner, there were no differences in scores given between autistic and non-autistic people, meaning that married autistic people were just as close to their partners as non-autistic people and the small to negligible effect sizes here emphasises the lack of substantial difference between the groups. This is in line with qualitative and mixed-methods work which has shown that autistic women, in particular, experience their intimate relationships as emotionally close and supportive [19, 57]. While long-term outcome work has shown that a minority of autistic people get married [16, 42], these samples are individuals originally diagnosed in the 1970s–1990s and who are therefore more likely to have co-occurring learning difficulties which will also impact their life outcomes. There are very few studies of the experiences of married autistic people, of any gender, with most scholarship on autism and marriage instead focussing on the marriages (and marriage breakdown) of parents of autistic children. Future work should consider the relationships of people diagnosed in adulthood, who are potentially more likely to have reached traditional social milestones, and how they and their partners adapt to their needs within the relationship.

The fact that this finding reinforces other work which shows that autistic people can have, maintain, and value close romantic relationships and friendships is supremely important. Traditionally, autism research has presumed that because autism is characterised by difficulties with social imagination and social relationships [7], autistic people may not want friends. This narrative has been thoroughly challenged in recent research with both autistic young people [20, 27, 58] and adults [57, 59], and the results from the URCS further undermine it. It may be that autistic people are likely to have one or two close friends or a single partner who they rely on for most of their social fulfilment, as has been seen in work with autistic women [19]. It is therefore logical that they rate a stable, long-term, intimate relationship more highly than non-autistic people who often have more diffuse social networks [53, 60], and more highly than best-friendships or dating relationships.

This intensity of feeling and social support from a few close relationships is not captured in measures such as the FQ, where in the very first question having ‘a few’ close friends is scored lower (2 points versus 5 points) than ‘one or two’ best friends, and later questions give greater points to socialising in group situations. This assumed hierarchy of ‘value’ to social interactions inherent in the FQ is based on seeing non-autistic social stereotypes as the norm, desirable, and the ‘best’ way to have friends. There is, however, no reduction in the value of the close relationships autistic adults have simply because they may have fewer of them, and researchers should be looking at whether autistic people are satisfied with the friendships they have rather than assuming that they are lesser because they do not always fit a non-autistic model. While currently there are no autism-specific friendship measures, we would argue that findings using the FQ are paired with qualitative work asking participants for greater insight into their experiences in future work, so as to avoid reinforcing the often-seen bias that autistic people do not want, or have, friendships [6]. The future development of an autism-specific friendship measure should be developed in partnership with a range of autistic people, in order to best reflect their experiences and priorities in friendship research.

## Limitations

Despite the importance of this replication study, there are some limitations. First, the data come entirely from self-report, including self-report of diagnoses. This means that we do not have independent verification of autism diagnoses, but the significantly higher AQ scores of the autism group suggest that we can be confident in typifying those participants who self-reported being autistic as genuinely being so. It is also important to recognise that many autistic adults, especially women and NBT people, face challenges in the formal diagnostic process [38, 61], and so, we have chosen to respect self-reported autistic identity. It should also be noted that there are recognised problems with the validity of the AQ, such as inadequate factor structure [62], a high proportion of false-negative results [63], and inherent biases [64]. Second, the groups were not matched on gender or employment status. This is to be expected, however, considering work showing that autistic people are more likely to be gender non-conforming than non-autistic people [40, 41] and that they can struggle to maintain full-time employment [39, 65, 66], and these differences are representative of the population. It may be that difficulties maintaining employment also limit opportunities to build friendships for autistic people, something which would be a valuable topic for future qualitative research. It is worth noting that there were far more women than men who took part, both autistic and non-autistic, and this may mean that the male sample is less representative than the female sample. Despite this, the male sample size is larger than that seen in many autism studies and is well-powered to detect group differences. Furthermore, there were very few non-autistic NBT people who took part in this study, despite active recruitment on the part of the team. This means that some of the conclusions about the nature of NBT people’s relationships must be treated with extreme caution, and future research which exclusively prioritises the potentially unique experiences of NBT people should be conducted. Third, not every participant chose to—or could—name a romantic partner or best friend to rate on the URCS. There were more autistic than non-autistic participants who did not complete the URCS, suggesting that even though those who did respond had very close nominated relationships, it may be more difficult for autistic people to make them in the first place, something which is not captured in this study. Future work would benefit from including qualitative portions to understand the experiences of this cohort in more detail. Fourth, the data presented here speaks only to the quantitative patterns observed in the relationships reported and cannot answer qualitative questions as to why those patterns exist. Future work should seek to do qualitative research with autistic people to explore why gendered patterns are different in this population to those seen in the non-autistic population.

## Conclusions

In conclusion, while our paper replicates the core finding of Baron-Cohen and colleagues’ original 2003 paper that autistic people score lower on the FQ than non-autistic people, it also expands and complicates their earlier findings. We found that there is a significant impact of gender on FQ scores for both autistic and non-autistic adults, with autistic women and NBT people scoring higher than autistic men, and non-autistic women scoring higher than other non-autistic groups. The inclusion of the URCS further elaborates the picture, showing that autistic people who have a romantic partner or best friend rate these relationships as closer than their non-autistic counterparts. Future work should seek to examine the differences between those who do and do not identify with their gender assigned at birth in terms of their relationships. Researchers should use our findings to complicate their conceptualisation of the relationships of autistic people, to challenge the dominant assumption that firstly, autistic people do not have meaningful relationships, and that secondly, all autistic people have the same relationship experiences regardless of gender. Gender plays an essential and acknowledged role in the lives of non-autistic people, and it should be considered equally important for those on the spectrum. It is important for clinicians and professionals to recognise that autistic people are capable of and interested in having friendships and romantic relationships and should be reassuring for autistic people and their families that those relationships can be just as good as those of non-autistic people.

## Supplementary information

**Additional file 1: Figure S1**. Supplementary Figure 1.

**Additional file 2: Figure S2**. Supplementary Figure 2.

**Additional file 3: Figure S3**. Supplementary Figure 3.

**Additional file 4: Figure S4**. Supplementary Figure 4.

## Data Availability

The datasets used in the current study are available from the corresponding author on reasonable request.
