# Supplementary figures and images for: The Friendship Questionnaire, autism, and gender differences: a study revisited
Source: Mol Autism. 2019 Nov 28;10:40. doi: 10.1186/s13229-019-0295-z (PMC6883660; doi:10.1186/s13229-019-0295-z)

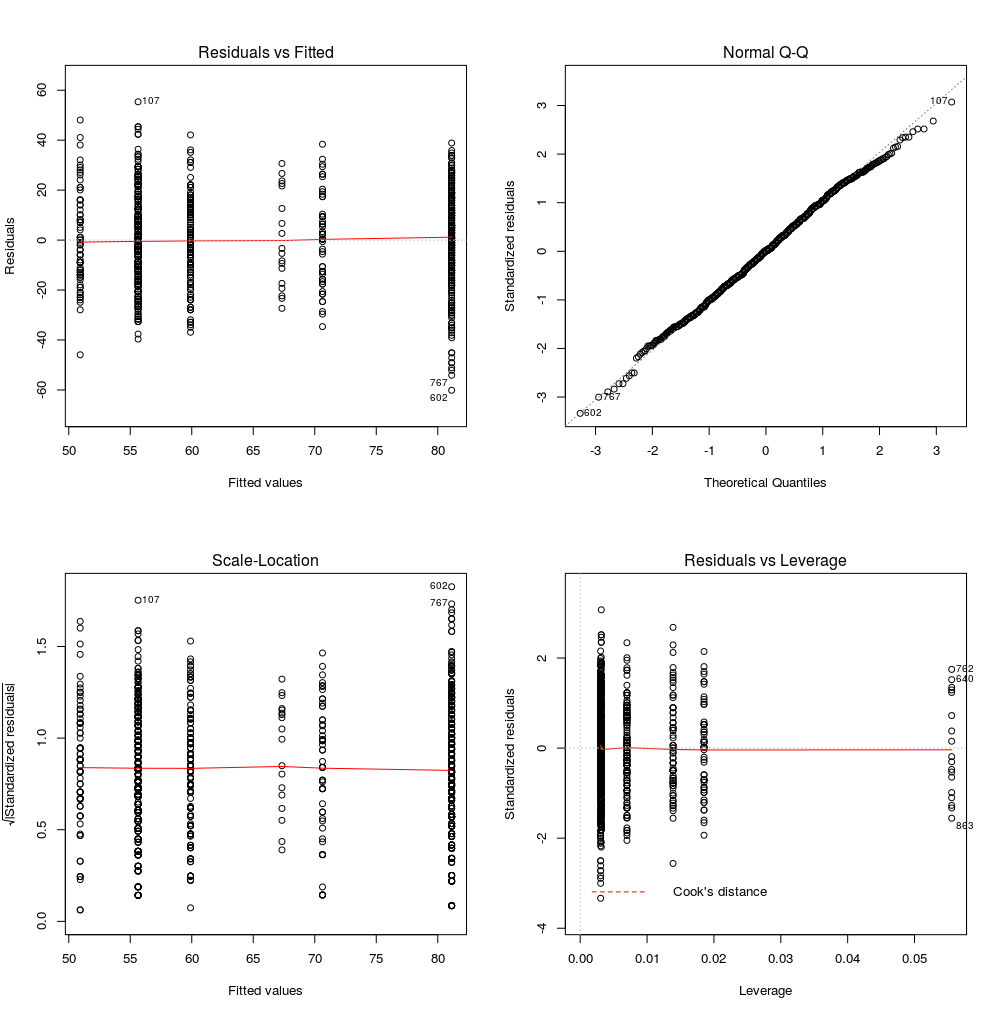

Supplement: Supplementary file 1 — Additional file 1: Figure S1. Supplementary Figure 1. [file 13229_2019_295_MOESM1_ESM.tiff]

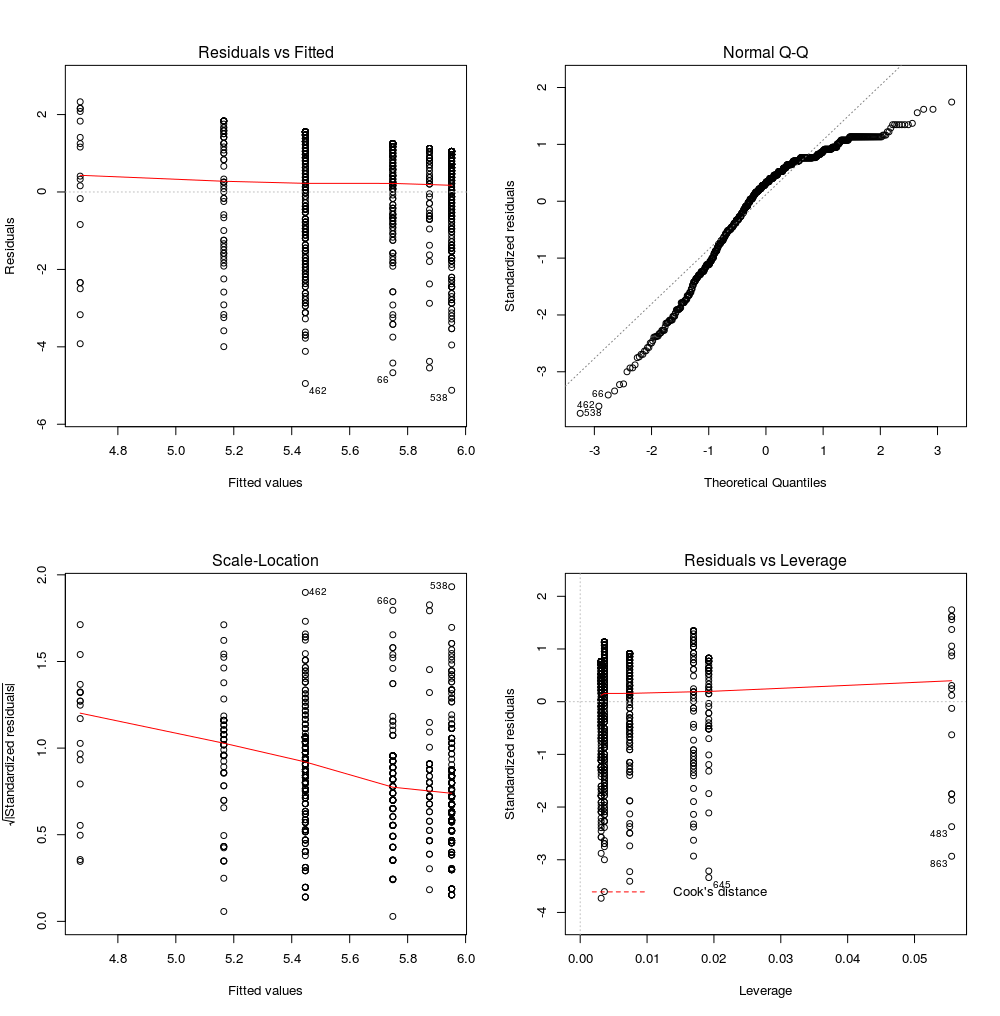

Supplement: Supplementary file 2 — Additional file 2: Figure S2. Supplementary Figure 2. [file 13229_2019_295_MOESM2_ESM.tiff]

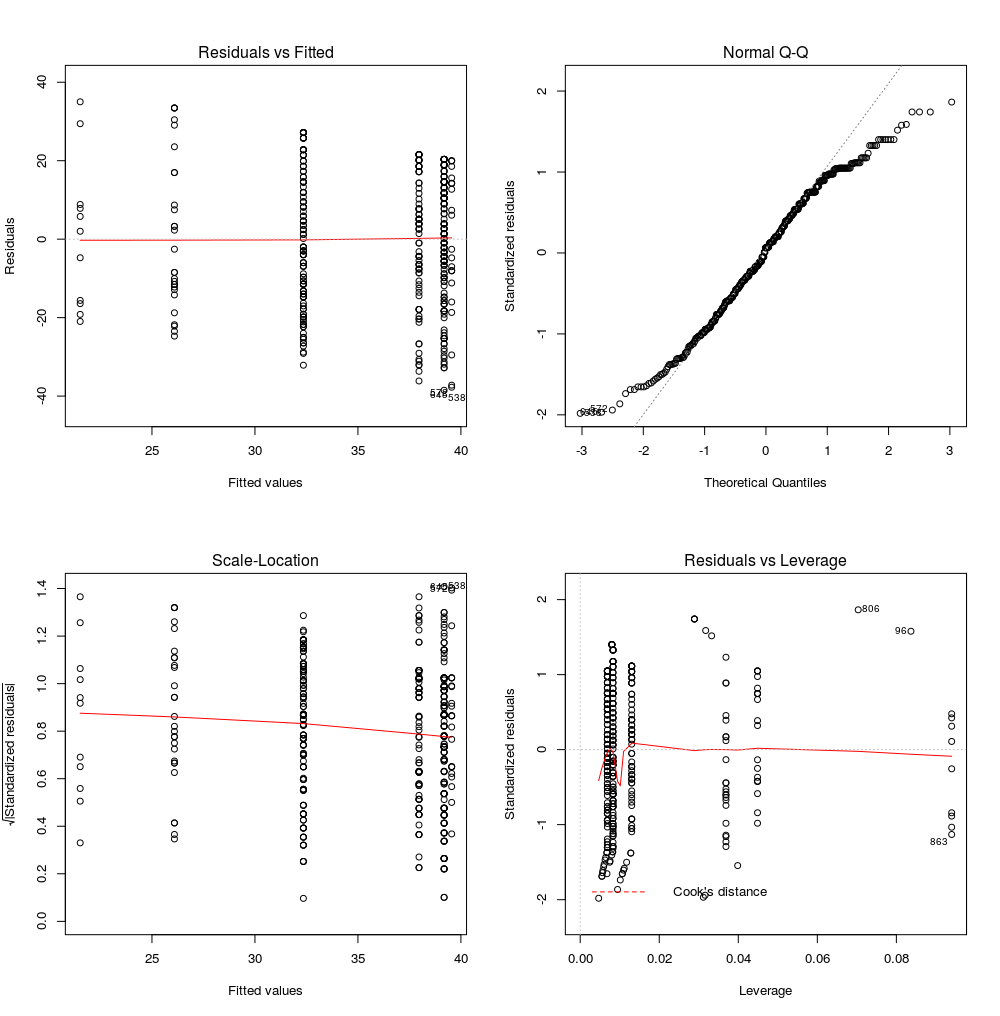

Supplement: Supplementary file 3 — Additional file 3: Figure S3. Supplementary Figure 3. [file 13229_2019_295_MOESM3_ESM.tiff]

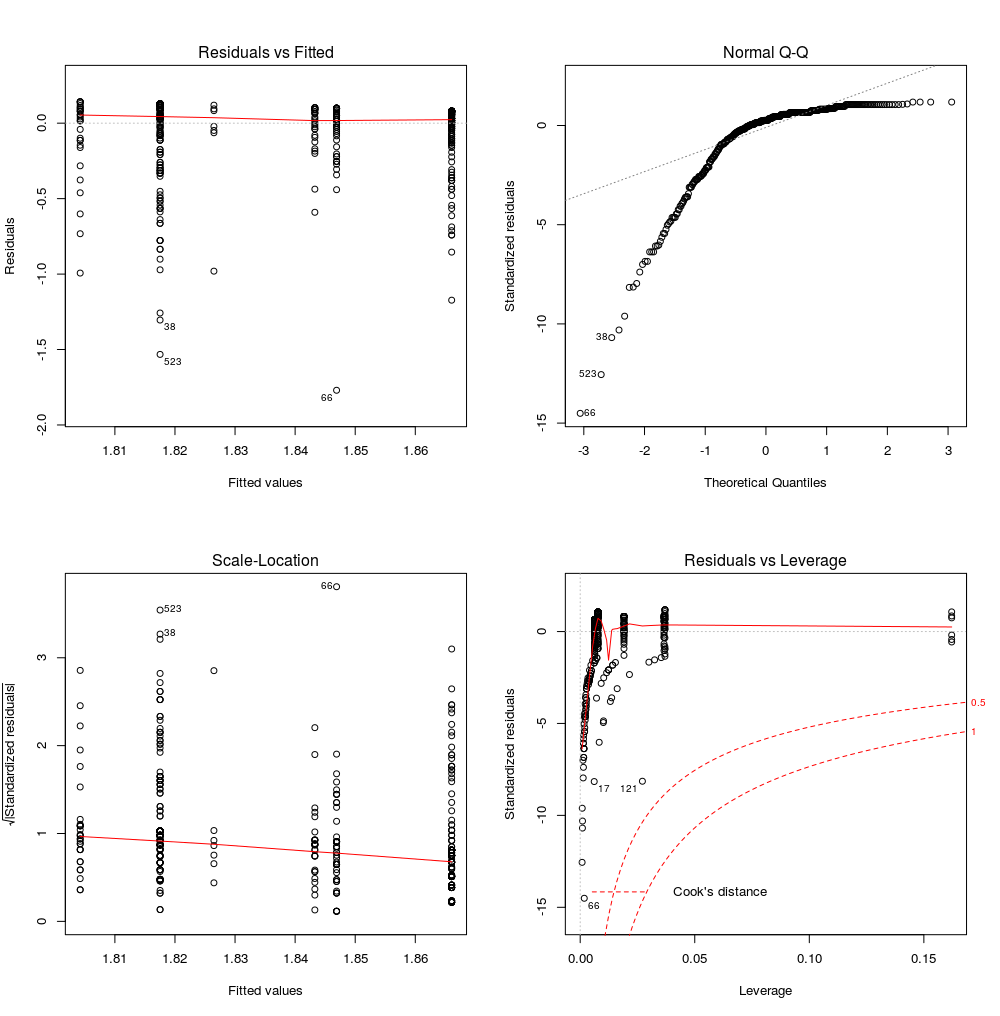

Supplement: Supplementary file 4 — Additional file 4: Figure S4. Supplementary Figure 4. [file 13229_2019_295_MOESM4_ESM.tiff]
